# Modulation of the biocompatibility of collagen/polyelectrolyte semi-IPN hydrogels with Zn-bioMOFs

**DOI:** 10.5599/admet.2074

**Published:** 2024-03-05

**Authors:** Martín Caldera-Villalobos, Jesús A. Claudio-Rizo, Denis A. Cabrera-Munguía

**Affiliations:** Facultad de Ciencias Químicas. Universidad Autónoma de Coahuila. Unidad Saltillo. Saltillo, Coahuila, 25280, México

**Keywords:** Wound healing, chronic wounds, wound dress, L-histidine, ZIF-8

## Abstract

**Background and purpose:**

In this study, we examined the impact of Zn-bioMOF structures on the physical and chemical characteristics as well as the *in vitro* biocompatibility of a matrix composed of semi-interpenetrating polymeric networks (semi-IPN) made from collagen and L-tyrosine-based polyelectrolytes.

**Experimental approach:**

We hydrothermally synthesized L-1, ZIF-8H Zn-bioMOFs, and the Zn-(L-His)_2_ complex, utilizing L-histidine, a bioactive amino acid, as a ligand. These metal-organic compounds primarily enhance the mechanical properties of the novel composite hydrogels through physical interactions such as hydrogen bonds and dipolar interactions. They also accelerate the gelation process. Composites containing Zn-bioMOFs exhibited greater biocompatibility than the collagen/polyelectrolyte matrix alone, as evidenced by cytotoxicity assays conducted with porcine fibroblasts, human monocytes, and RAW 264.7 cells. Furthermore, the evaluated materials did not exhibit hemolysis. We investigated the influence of Zn-bioMOFs on cell signaling by measuring the levels of crucial cytokines involved in the healing process, such as MCP-1, TGF-β, IL-10, and TNF-α secreted by human monocytes.

**Key results:**

The composite with Zn(L-His)2 promoted the secretion of MCP-1, TGF-β, and IL-10, while a decrease in TNF-α secretion was observed with the composite containing ZIF-8H. Zn-bioMOFs enhanced certain aspects of the biomedical and physicochemical properties of the composite hydrogels.

**Conclusion:**

Although the overall performance of the tested materials did not differ significantly, it is worth noting that the presence of Zn-bioMOFs in biopolymeric hydrogels modulated the metabolic activity of cells important for healing and their cytokine signaling, leading to improved biomedical performance.

## Introduction

Metal-organic frameworks (MOFs) are novel hybrid organic-inorganic supramolecular materials characterized by well-structured networks formed by organic electron-donating linkers and metal cations. They represent an expanding group of versatile nanoporous substances with a wide range of applications, including but not limited to separation technology, catalysis, drug delivery, electronics, optics, and sensing [1]. Among these, MOFs based on zinc (Zn), iron (Fe), and zirconium (Zr) have received significant attention for their potential in biomedical uses [2]. Zn-based MOFs are particularly noteworthy due to their nontoxic nature of Zn^2+^ ions, antimicrobial properties, and established track record in dermatology as a healing and skin-moisturizing agent [3]. Zn-MOFs hold promise as materials for various applications such as drug carriers, nanoencapsulation, magnetic resonance imaging agents, nitric oxide storage and delivery, chemosensors, biosensors, and more [4].

Zn-MOFs can be produced using diverse ligands, but it is important to prioritize their resistance to hydrolytic degradation when considering their use in biomedical applications. In contrast to MOFs formed with carboxylic ligands, those incorporating nitrogen-based ligands demonstrate improved stability [5]. Zeolitic imidazolate frameworks (ZIFs) belong to a subset of MOFs characterized by the association of tetrahedral transition metal ions with linkers derived from imidazolate compounds [6]. ZIF-8 and its biocompatible counterparts find utility in various fields, including biosensors, the treatment of cancer and bacterial infections, wound healing, and biocatalysts [6]. Additionally, it was acknowledged that ZIF-8 coatings can potentially serve as advantageous materials for enhancing the surface properties of titanium implants [7].

Certain ligands that contain nitrogen, such as amino acids, peptides, nucleobases, vitamins, or active pharmaceutical ingredients, are derived from natural sources and possess bioactive properties. MOFs formed using these ligands constitute a specific subclass referred to as bioMOFs [8-9]. These bio-based ligands offer enhanced biological compatibility compared to conventional MOFs, opening up numerous possibilities for a broad range of biological uses [10]. Zn-bioMOFs have been successfully generated using nucleobases like cytosine and guanine [11]. Amino acids are chiral ligands with a stronger affinity for metal ions with higher valence states. However, the inherent chirality of amino acids limits their ability to form three-dimensional networks [12]. Consequently, the occurrence of BioMOFs constructed solely from amino acids is quite rare. Most BioMOFs are constructed by combining or modifying amino acids with other organic ligands [13]. Nevertheless, there have been reports of Zn-bioMOFs synthesized from glutamic acid [14], aspartic acid [15], and serine [16]. The synthesis of MOFs using naturally occurring components such as amino acids may be preferable to those derived from non-biological sources. This approach is particularly successful for Zn-MOFs incorporating phenylalanine and tyrosine derivatives [17].

In recent investigations, we assessed how Zn(Atz)(Py) affects the biomedical performance of a hydrogel matrix composed of collagen, guar gum, and polyurethane. Our findings showed that this Zn-MOF significantly boosted the viability of human monocytes and porcine fibroblasts when cultured in contact with the composite hydrogel. Additionally, Zn(Atz)(Py) played a role in modulating cellular signaling by stimulating the secretion of the cytokine MCP-1 while maintaining the secretion of TNF-α and TGF-β within normal ranges. Furthermore, Zn(Atz)(Py) exhibited enhanced capabilities in inhibiting bacterial growth and in facilitating drug release [18]. Additionally, our research team reported enhanced biocompatibility of collagen-polysaccharide hydrogels by incorporating a Zn-MOF containing L-tryptophan or L-phenylalanine ligands. The remarkable adhesion properties and cell adaptability to these hydrogels suggest their potential as effective materials in biomedical applications, including wound dressing and tissue engineering [19]. Based on the information provided, the objective of this study was to assess how effectively Zn-bioMOFs can influence the biological properties of hydrogel matrices comprised of collagen-amino acid-based polyelectrolyte. In this investigation, we tested two MOFs utilizing the L-histidine ligand and compared the outcomes with those of the Zn(L-His)_2_ complex. This comparison aimed to determine whether the reticular structure of coordination polymers offers greater advantages for biomedical applications.

## Experimental

### Materials

L-tyrosine (L-Tyr), L-histidine (L-His), formaldehyde, sodium hydroxide, ethanol absolute, acetone, zinc nitrate hexahydrate, 1,3,5-benzenetricarboxylic acid (H_3_BTC), 2-methylimidazole (2mim), ammonium hydroxide, rhodamine, sodium chloride, potassium chloride, dibasic sodium phosphate, monobasic potassium phosphate, hexamethylene diisocyanate, glycerol ethoxylate (Mn ≈ 1000 g mol^-1^), and 3-(4,5-dimethyl-2-thi-azolyl)-2,5-diphenyl-2H-tetrazolium bromide (MTT) were acquired from *Sigma Aldrich Co.* in their original condition and employed them without further modifications.

### Polymer precursors

Collagen (with molecular weights of approximately 230 kDa for α1 and 110 kDa for α2) was obtained from porcine dermal tissue through enzymatic hydrolysis using pepsin (at a concentration of 1000 ppm in a 0.01 M HCl solution) while continuously stirring for 72 hours at room temperature [20]. The concentration of collagen was set to 6 mg mL^-1^, then stored at 4 °C until it was ready for use.

Water-based polyurethane used for collagen crosslinking was formed through the reaction of glycerol ethoxylate with hexamethylene diisocyanate at 80 °C for 2 hours, with a molar ratio of 3:1 for NCO:OH. To block the isocyanate end-groups of the prepolymer, a sodium metabisulfite solution (40 wt.%) was introduced, and the mixture was stirred at 40 °C for 2 hours to ensure the reaction's completion [21].

The amino acid-based polyelectrolyte was produced through a polycondensation process involving L-Tyr and an excess of formaldehyde (at a 1:2 mol/mol ratio). This reaction was catalyzed by NaOH (10 % mol) in the presence of water as the solvent [22]. The reaction mixture was continuously stirred using a magnetic stirrer at 60 °C for 24 hours. Subsequently, the resulting yellow product was purified through ethanol and acetone treatment. RMN ^1^H (400 MHz, D_2_O) *δ* / ppm: 7.18-6.45 (brs, 2H, Ar-H), 4.74-4.38 (brs, 1H, CH_2_), 4.05-3.22 (brs, 2H, CH), 3.18-2.54 (brs, 1H, CH). FTIR (ATR, cm^-1^): 3230 (νArO-H), 2930 (*ν*_asym_ CH_2_), 2861 (*ν*_sym_ CH_2_ str), 1579 (*ν*_asym_ CO_2_^-^), 1479 (νC=C), 1395 (*ν*_sym_ CO_2_^-^), 1223 (*ν* C-O), 1021 (νC-O).

### Synthesis of Zn-bioMOFs

The compound [Zn_4_(btc)_2_(Hbtc)(L-His)_2_(H_2_O)_4_]∙1.5H2O (referred to as L-1) was prepared through a hydrothermal synthesis method following the protocol described by He *et al.* [23]. A blend of Zn(NO_3_)_2_×6H_2_O (weighing 5.2682 g), H_3_BTC (3.2002 g), L-His (2.2905 g), and 50 mL of water underwent magnetic stirring at room temperature for 30 minutes. Subsequently, this mixture was transferred into a Teflon-lined autoclave with a pH of 4.4 and heated in an oven at 130 °C for 96 hours. After cooling, the resulting crystalline product was separated by filtration, washed with water, and dried at 60 °C for 24 hours.

The synthesis of zeolitic imidazolate framework modified with L-His (ZIF-8H) followed the standard protocol used for synthesizing ZIF-8. However, in this case, a partial substitution of the ligand 2mim with L-His was introduced, as described in the provided reference [24]. To achieve this, 1.0204 g of L-His and 1.0526 g of 2mim were dissolved in 30 mL of deionized water. The resulting mixture underwent magnetic stirring for 5 minutes, following which 1.8469 g of Zn(NO_3_)_2_×6H_2_O, previously dissolved in 30 mL of water, was introduced. Subsequently, to attain a pH of 7.6, 1.1 mL of NH_4_OH (28 wt.%) was added. The solution was continuously stirred for 24 hours, resulting in the formation of a colorless precipitate. The remaining unreacted components were separated through centrifugation. Lastly, ZIF-8H was subjected to drying at 60 °C for 24 h.

To produce the Zn(L-His)_2_ complex, we began by dissolving 5.2682 g of Zn(NO_3_)_2_×6H_2_O in 25 mL of water. Subsequently, 4.581 g of L-His was introduced, pre-dissolved in 25 mL of water. To attain a pH of 7.4, NH_4_OH (28 wt.%) was used for pH adjustment [25]. The process was conducted at ambient room temperature while employing magnetic stirring for 24 hours. The resulting colorless product underwent purification through centrifugation. Subsequently, it was subjected to drying at 60 °C for 24 hours.

Zn-bioMOFs underwent characterization through several techniques, including Fourier transform infrared spectroscopy (FTIR), wide-angle X-ray scattering (WAXS), and scanning electron microscopy (SEM). FTIR spectra were collected using a Perkin-Elmer Frontier spectrophotometer with an ATR accessory. To assess crystallinity, WAXS analysis was performed using an Anton Paar SAXS-Emc2 diffractometer equipped with a Cu Kα X-ray source (*λ* = 15.4 nm). The morphology of the MOFs was observed via SEM, utilizing a JEOL JSM-6510LV/LGS microscope operating at 15 kV. To prevent the buildup of electrostatic charge, samples were coated with graphite.

**Figure 1.**
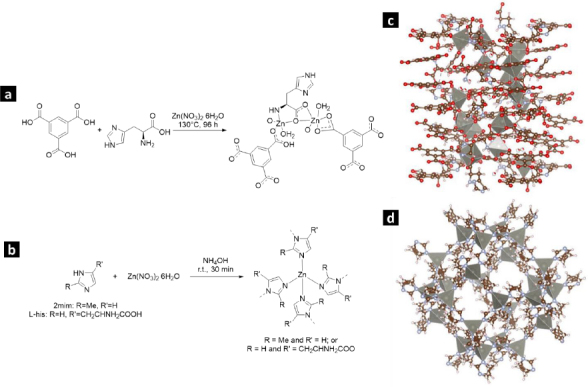
(a) Synthesis of L1; (b) Synthesis of ZIF-8H; (c) Crystal structure of L1; (d) Crystal structure of ZIF-8.

Figures 1(a-b) illustrate the production process of Zn-bioMOFs incorporating the L-His ligand. In Figures 1c and 1d, we present the crystal structures of L-1 and ZIF-8, respectively.

### Synthesis of composite hydrogels.

**Figure 2.**
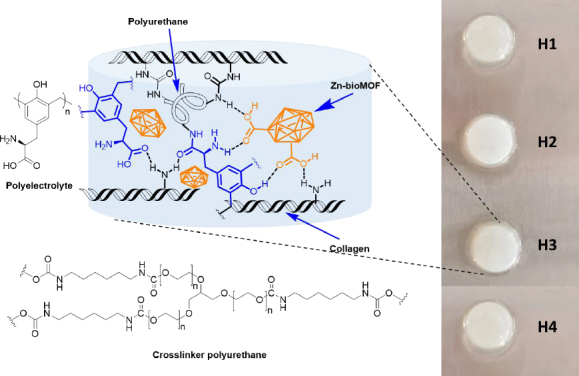
Schematic representation of the chemical structure of composite hydrogels with semi-IPN matrixes.

Dispersions containing Zn-MOFs (0.1 wt.%) in aqueous collagen solutions (6 mg mL^-1^) were prepared in advance. Hydrogels were then formed using 24-well culture plates as molds. In each well, 1 mL of either collagen solution or MOF dispersion was combined with 15 μL of polyurethane crosslinker (at a concentration of 15 wt.%). This process was carried out while maintaining a temperature of 4-5 °C, typically on an ice bath. Subsequently, an appropriate volume of polyelectrolyte solution (2 wt.%) was added to each formulation. After thorough mixing, the pH was adjusted to 7 by adding 200 μL of phosphate buffer solution (PBS-10X). Finally, the crosslinking process was conducted at 37 °C for 24 hours. The quantities used for preparing the composite hydrogels are presented in Table 1, and Figure 2 displays a schematic representation of their chemical structure.

**Table 1.** Labels and compositions for composite hydrogels.

Key	Composing material	Amount, μg		
		Collagen	Polyurethane	Polyelectrolyte
H1	None	6	0.9	0.6
H2	L-1(60)	6	0.9	0.6
H3	ZIF-8H (60)	6	0.9	0.6
H4	Zn(L-His)_2_ (60)	6	0.9	0.6

The water absorption capacity was assessed through a gravimetric method. The cross-linking of semi-IPNs was examined using the ninhydrin reaction [26]. The physicochemical properties of the composite hydrogels were analyzed using FTIR, WAXS, SEM, and oscillatory rheometry. The storage modulus (*G*') of the hydrogels was determined via low-amplitude oscillatory rheometry utilizing an Anton-Paar Physica MCR 301 rheometer. The experiments were conducted at 37 °C using a plate-plate geometry with a diameter of 40 mm. Measurements were executed with a 10 % strain to observe the viscoelastic properties in the dynamic behavior of the hydrogels.

The kinetics of gel formation in the composite matrix were investigated using turbidimetric analysis. Samples measuring 200 μL from the polymerizable mixtures were placed in a 96-well microplate, and their absorbance at 550 nm was measured at 37 °C every 30 seconds for a total duration of 90 minutes using the ThermoScientific Multiskan Sky spectrophotometer. Kinetic parameters such as nucleation time (*t*_lag_) (the phase with low absorbance values), average gelation time (*t*_1/2_) (the time at which half of the maximum absorbance value is achieved), and gelation rate (*S*) (the slope of the linear region of the curve) were determined from the collected data.

### Evaluation of the biocompatibility in vitro

#### Cell cultures

To prepare the monocyte culture, a human blood sample underwent centrifugation at 4000 rpm for 30 minutes. Following this, the supernatant (plasma) was removed, and the cells settled at the interface of the solid-liquid phase were collected. These cells were then placed in a conical tube and washed with PBS 1X, after which they were centrifuged for 10 minutes, and the supernatant was discarded. Subsequently, the collected cells were transferred to a Falcon tube, and RPMI (Roswell Park Memorial Institute) culture medium was added at a ratio of 10.44 g L^-1^ of solution along with 20 μL of penicillin-streptomycin antibiotic. The cells were incubated at 37 °C and quantified using a Neubauer chamber to achieve cell cultures with a density of 30,000 cells per mL.

To establish the fibroblast culture, cells were extracted from a section of porcine skin obtained from a recently slaughtered animal provided by the municipal slaughterhouse. The porcine skin and subcutaneous fat, totaling 15 g, were carefully collected. Subsequently, three washing steps were carried out using 25 mL of sterile PBS 1X for 10 minutes each, facilitated by gentle vortex stirring. Following the washes, the tissue was retrieved and finely rinsed in 100 mL of sterile PBS 1X. A portion of 45 mL from this mixture was transferred to a Falcon tube. To initiate the proteolytic degradation of the extracellular matrix, 5 mL of trypsin 1X, 500 μL of collagenase solution (14 units), and 10 μL of penicillin-streptomycin antibiotic were added. The mixture was stirred for 1 hour at 37 °C. Subsequently, the medium was rinsed again and then centrifuged for 5 minutes to collect the cell pellet. Finally, to prepare the culture medium, the harvested cells were blended with 1.044 g of DMEM culture medium (Dulbecco's Modified Eagle's Medium), along with 10 μL of antibiotics, and adjusted to a final volume of 50 mL using sterile PBS 1X. The cells were incubated at 37 °C and quantified using a Neubauer chamber to achieve cell cultures with a density of 30,000 cells per mL.

RAW 264.7 cells (ATCC TIB-71) were cultivated in DMEM containing 10 vol.% fetal bovine serum (FBS) and 1 vol.% antibiotics (penicillin-streptomycin). The cells were incubated at 37 °C in an environment with 5 % CO_2_.

#### Cell viability

The MTT assay was employed to assess the metabolic activity of human monocytes, porcine dermis fibroblasts, and RAW 264.7 cells when they were in contact with the composite hydrogels. PBS-1X served as the positive control. Cell viability below 60 % was regarded as indicative of cytotoxicity. Samples containing RAW cells were cultured at 37 °C in a 5 % CO_2_ atmosphere, while human monocytes and fibroblasts were cultured under normal air conditions.

#### Proliferation of RAW 264.7 cells

RAW 264.7 cells stained with rhodamine B, cultured near semi-IPN hydrogels for 48 hours at 37 °C in a 5 % CO_2_ atmosphere, were examined using a VELAB VE-146YT fluorescence microscope equipped with a 40× objective. Excitation of the samples was achieved using a green LASER at a wavelength of 532 nm.

#### Hemolysis assay

Hemocompatibility of the hydrogels was assessed through a hemolysis assay, which involved measuring the release of hemoglobin following the disruption of erythrocyte cell membranes. In this procedure, erythroncyte samples that had been previously purified in Alsever's solution (112 μL) were combined with 150 μL of the leachate extracted from the composite hydrogels and 1728 μL of Alsever's solution. For control purposes, Alsever's solution and deionized water were employed as the negative control (0% hemolysis) and positive control (100% hemolysis), respectively. These mixtures were then incubated at 37°C with orbital stirring (250 rpm) for 30 minutes. Following incubation, the samples were subjected to centrifugation at 3000 rpm, and aliquots were collected from the supernatant. The absorbance of these samples was measured at 415 nm using a ThermoScientific Multiskan Sky spectrophotometer.

### Evaluation of the cell signaling by enzyme-linked immunosorbent assay (ELISA)

The levels of certain key cytokines associated with the wound healing process, namely monocyte chemoattractant protein 1 (MCP-1), transforming growth factor-β (TGF-β), interleukin 10 (IL-10), and tumor necrosis factor-α (TNF-α), were quantified using ELISA. These assays were conducted on human monocyte cultures, utilizing kits obtained from Invitrogen and following the supplier's instructions.

## Results and discussion

### Characterization of MOFs

**Figure 3.**
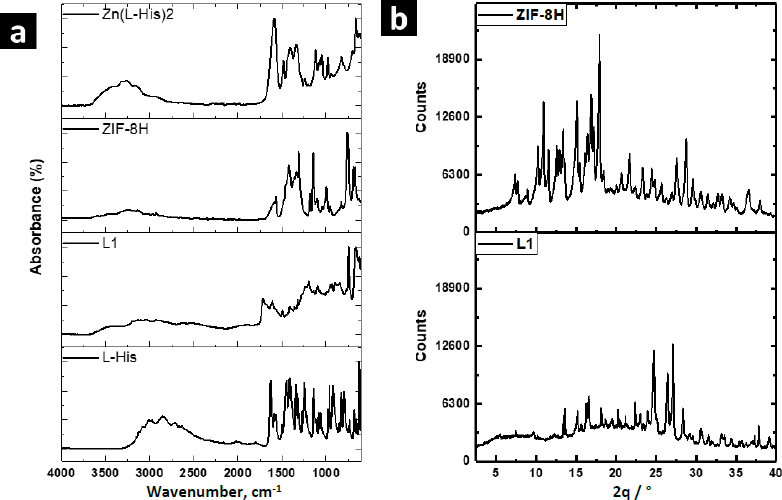
(a) FTIR spectra of Zn-bioMOFs; (b) XRD patterns of Zn-bioMOFs.

Zn-MOFs underwent characterization using FTIR, WAXS, and SEM. Figure 3a displays the FTIR spectra of the L-His ligand and their coordination polymers. L-1 was synthesized by self-assembling H_3_BTC and L-His ligands with the Zn^2+^ ion. The band at 1608 cm^-1^ was attributed to the νC=C vibration originating from the aromatic ring of BTC, while the bands at 3099 and 3040 cm^-1^ were associated with the =C-H stretching of the aromatic ring. Additionally, the bands at 1712 and 1672 cm^-1^ were assigned to the free and coordinated carboxylic acid groups, respectively. The band at 3133 cm^-1^ was attributed to the free -OH groups from non-coordinated carboxylic acid groups. Some bands related to L-His were observed at 1493 and 1410 cm^-1^, corresponding to the stretching vibration of the imidazole ring. Furthermore, the band at 2927 cm^-1^ was assigned to the asymmetric stretching vibration of the methylene group in the amino acid. Finally, the band at 3449 cm^-1^ was linked to the stretching vibration of the free amino groups in L-histidine.

The FTIR spectrum of ZIF-8H exhibited a broad band originating from the stretching vibration of the imidazole ring, located at 1417 cm^-1^. At 1560 cm^-1^, there was a band attributed to the carbonyl stretching of L-His, and this band shifted to a lower frequency due to the coordination involving oxygen atoms. Similarly, in the spectrum of the Zn(L-His)_2_ complex, the stretching vibration band of the imidazole ring appeared at 1406 cm^-1^, and the coordinated carbonyl band was observed at 1580 cm^-1^. In all three coordination products, there was an absorption band at 690 cm^-1^, which originated from the stretching of the Zn-O bond. Absorption bands related to the Zn-N bond were not detected, as they typically appear at lower frequencies. It is worth mentioning that when L1 incorporates aromatic ligands, the intensity of signals observed is comparatively reduced compared to other Zn complexes that feature L-histidine and imidazole as ligands. This suggests that L1 consists of planes closely packed through coordination interactions, which in turn restricts the vibration of the linkages.

The XRD patterns confirmed the crystalline nature of coordination polymers L-1 and ZIF-8H, which is evident from strong diffraction peaks. In the case of L-1, the main peaks were detected at 10.9, 15.1, 16.8, and 18.0°, whereas the most prominent peaks for ZIF-8H were observed at 24.7, 26.3, and 27.1°. In contrast, the structure of the complex Zn(L-His)_2_ appeared entirely amorphous, characterized by a broad halo pattern without any discernible diffraction peak (the pattern is not shown). As previously mentioned, the challenge of constructing MOFs exclusively from amino acids stems from the inherent chirality of these molecules. The high rotation of the chiral carbons in the amino acids as L-His generates disordered coordinated structures, preventing the generation of structures with molecular packing, and not appreciating the generation of crystalline cells. However, the Zn-bioMOFs L-1 and ZIF-8H, when including rigid ligands, generate metal-organic cloisters with a characteristic packing order; in the case of L-1, which has aromatic ligands, it generates laminar plates with defined crystallinity, and for ZIF-8H metal-organic tetrahedral networks are generated with a well-organized orientation and molecular arrangement. Examining the intensities of the XRD signals in the Zn-bioMOFs, it is important to highlight that ZIF-8 exhibits stronger signals compared to L-1. This observation validates a higher likelihood of molecular accommodation within ZIF-8, aligning with the vibrational characteristics observed in FTIR.

**Figure 4.**
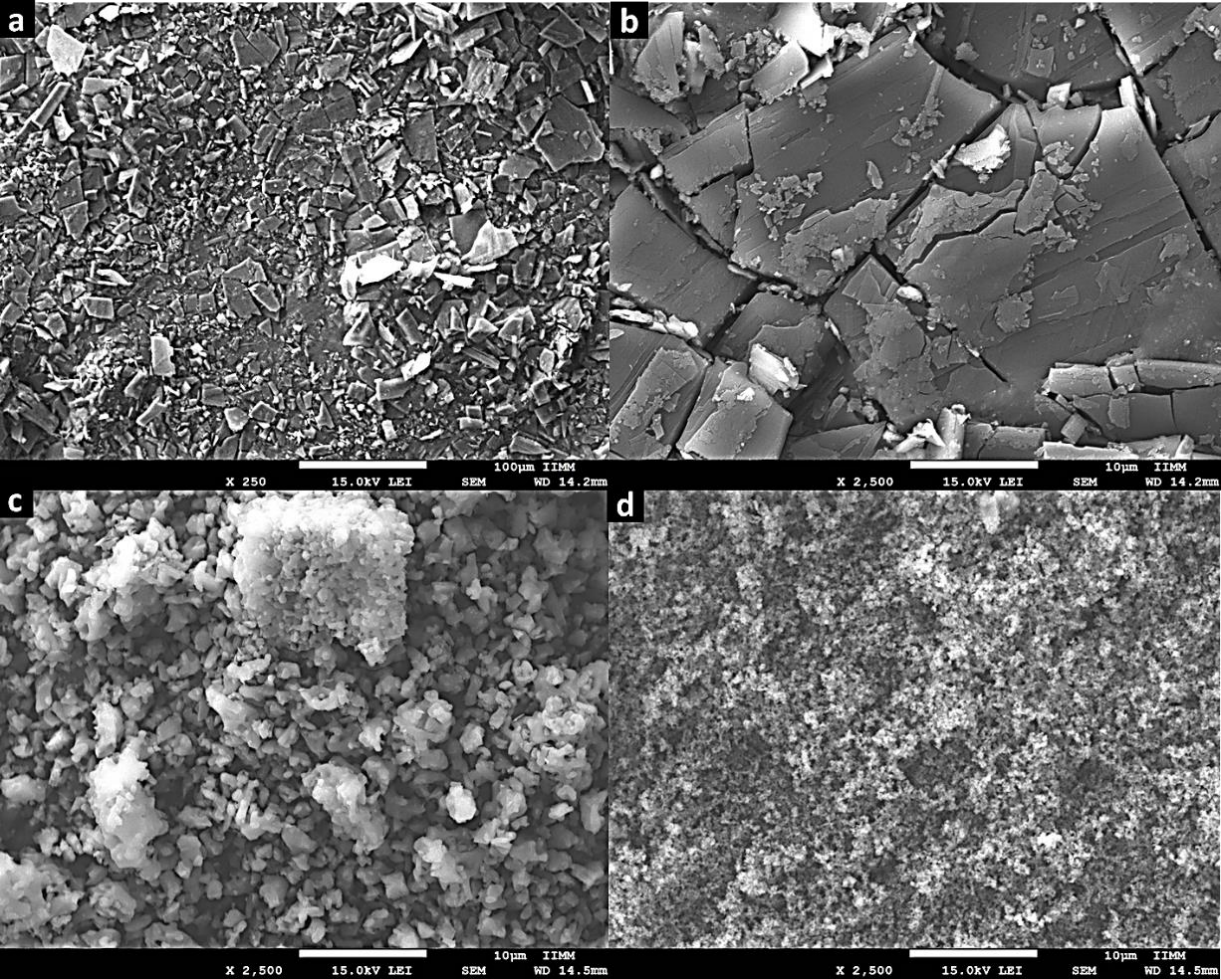
SEM Image of (a) and (b) L-1; (c) ZIF-8H and (d) Zn(L-His)_2_.

The SEM technique was employed to examine the morphology of Zn-MOFs. As depicted in Figure 4(a-b), images of L-1 display particles characterized by an irregular shape and a wide size distribution. In contrast, the morphology of ZIF-8H comprises particles with a dendritic growth pattern. Lastly, Zn(L-His)2 is composed of quasi-spherical particles that form multiple agglomerates. As substantiated by XRD analysis, L-1 tends to produce flat structures with well-defined crystallinity. In contrast, ZIF-8H tends to form agglomerates displaying a tetrahedral geometry with a high degree of crystallinity.

Finally, the Zn(L-His)2 complex generates rugged surface features due to electrostatic interactions among particles, resulting in an amorphous surface texture. The chemical composition and structural characteristics of each Zn-organic complex will play a crucial role in shaping the structural and biological properties of the composite hydrogels formed alongside collagen and the L-Tyr-based polyelectrolyte. These outcomes will be elaborated upon and discussed in subsequent sections. The organization of Zn^2+^ ions into flat, tetrahedral, or amorphous structures will have a significant impact on their biofunctionality, particularly in their ability to modulate the metabolism of vital cells involved in the wound-healing process. Additionally, these physicochemical interactions will directly influence cell signaling, which is essential for the production of cytokines that regulate the healing process.

### Characterization of composite hydrogels

Table 2 shows some physicochemical characteristics of composite hydrogels. They showed a high swelling capacity (>3000 %) and thus, they are considered super-absorbent materials. This could be due to the semi-interpenetration with the polyelectrolyte, which is a highly hydrophilic polymer with -OH, -COO-, and NH_2_ pendant groups, able to form hydrogen bonds retaining water molecules in the reticulated structure of the hydrogel. Significant outcomes result from the comparison of the swelling properties between H1 and H4. This comparison reveals that the Zn(L-His)_2_ complex, which is amorphous and fully dispersed within the collagen-tyrosine polyelectrolyte matrix, has an enhanced capacity for water absorption. This heightened water capture ability is attributed to its ability to form hydrogen bonds more freely with the metal-organic structure. Furthermore, the presence of ZIF-8H and L-1 crystalline structures within the semi-IPN polymer matrix also contributes to increased swelling, although to a lesser extent than H4. This is because water uptake is constrained within the clusters exhibiting a crystalline structure, characterized as lamellar and tetrahedral for L-1 and ZIF-8H, respectively.

**Table 2.** Physicochemical characteristics of composite hydrogels.

Hydrogel	Swelling degree, %	Crosslinking degree, %	*t*_lag_ / min	*t*_1/2_ /min)	*S* / min^-1^
H1	3009±279	48±2	7.9±0.1	9.9±0.1	0.0431±0.01
H2	3263±146	34±1	10.0±0.1	12.0±0.1	0.0927±0.01
H3	3203±258	34±2	9.9±0.1	11.9±0.1	0.1266±0.01
H4	3309±342	8.9±0.6	10.1±0.1	11.8±0.1	0.0870±0.01

Composite hydrogels showed low reticulation. We observed that the addition of Zn-MOFs decreased the chemical crosslinking of the collagen-polyelectrolyte-polyurethane matrix. In the H4 system, there is a correlation between swelling and the crosslinking ratio, with higher swelling observed when the crosslinking is at its lowest. This phenomenon can be attributed to the presence of hydrophilic groups found in histidine's imidazole rings and in the aromatic rings of BTC. These groups hinder the crosslinking of the polar segments within the biopolymer chains. In the L4 system, there are stronger repulsive interactions that don't contribute positively to polymer cross-linking. In contrast, in H2 and H3, the reduction in crosslinking isn't as significant when compared to L1. This can be explained by the fact that crystallinity is more prominent in ZIF-8H and L-1, leading to a balance between hydrophobic and hydrophilic interactions that facilitate both physical and chemical crosslinking of the polymer.

**Figure 5.**
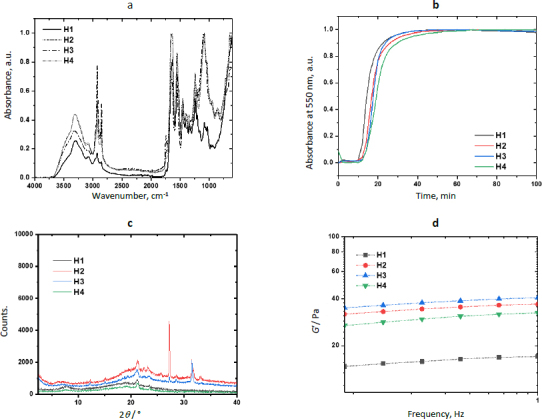
(a) FTIR spectra of composite hydrogels; (b) Kinetic profiles for the crosslinking reaction obtained by turbidimetry; (c) XRD patterns obtained by WAXS; (d) Plots of oscillatory rheometry.

Figure 5a shows results from the physicochemical characterization of hydrogels. The FTIR spectra of H1 and the composites H2-H4 showed the typical bands of Amide I (*ν*C=O) and Amide II (δN-H) in 1630 and 1544 cm^-1^ due to the polypeptide backbone of collagen. The band at 1742 cm^-1^ was attributed to the *ν*C=O vibration of the urea groups formed during the reticulation with the polyurethane. The intensity of this band increased in the spectrum of H2, showing that ZIF-8H increases the physical reticulation of the collagen matrix. The presence of the polyelectrolyte in the semi-IPN matrix was confirmed by the band of the ArO-H stretching observed at 3303 cm^-1^. A broad absorption band appeared in the spectrum of composites in 1092 cm^-1^ due to the addition of Zn-MOFs. This is evidence of the physical reticulation produced by MOF particles through secondary bonds. The increment in the signal intensity linked to the C-O bond at approximately 1250 cm^-1^ in the surfaces containing ZnMOFs can be attributed to a reduction in the semi-interpenetration process of collagen chains and L-Tyr-based polyelectrolyte. This reduction occurs because the repulsion effects caused by the non-polar regions within the metal-organic structures do not contribute favorably to the physicochemical crosslinking of the hydrogel composites. As a result, larger interpolymeric spaces are created, which tend to trap water molecules and, consequently, enhance the swelling properties.

MOFs are well known for their catalytic activity. Figure 5b shows the kinetic profiles of the reticulation reaction obtained by turbidimetry and Table 2 shows the kinetic parameters for each composite. Results showed that the evaluated Zn-bioMOFs increase the duration of the nucleation step (*t*_lag_) but increase the reticulation rate (*S*) involving the reaction between primary amino and isocyanate groups to yield the semi-IPN networks. From the sigmoidal curves, it was observed that semi-IPN hydrogels are completely formed after 50 min (plateau stage). The increase in nucleation time is connected to a delay in the semi-interpenetration process of the polymers. During this delay, hydrophobic interactions with polar groups hinder the acceleration of collagen nanofibril arrangement, resulting in the formation of larger diameter fibers being postponed. Once this initial barrier to fibrillar nucleation is overcome, the MOF's chemical structure becomes directly linked to the rate at which larger fibers are generated through the collagen fibrillogenesis process. The ZIF-8H MOF expedites this process. This acceleration can be attributed to the tetrahedral arrangements characteristic of the ZIF-8H MOF, which promote swift entanglement of fibrils. This suggests a catalytic role for ZIF-8H MOF in the collagen fibrillogenesis process, as collagen crosslinks with polyurethane and semi-interpenetrated polyelectrolytes. Additionally, it is noteworthy to highlight that the ZnMOF with the most pronounced crystalline structure demonstrates the most efficient catalytic activity during the collagen fibrillogenesis process. This hydrogel composite formulation holds potential for applications in healing patches designed for situations where there is a need for accelerated collagen fibrillogenesis within wound beds.

Figure 5c presents the scattering pattern of H1, showing an amorphous halo with a scattering peak at 2*θ* = 21.2°, showing that the polymer matrix has very low crystallinity. The scattering patterns of H2 and H3 show the aforementioned halo and a peak 21.2°. However, the patterns of H2 show two additional strong and sharp peaks at 2*θ* = 27.3 and 31.4°, which were previously observed in the scattering pattern of L-1. Similarly, the scattering pattern of H3 shows a peak at 31.4°, which was observed in the pattern of ZIF-8H. The pattern of H4 did not show any additional peak due to the amorphous nature of Zn(L-His)_2_. The incorporation of L-1 into the collagen-polyelectrolyte matrix leads to the formation of zones with elevated crystallinity. This outcome arises from L-1's composition, which includes lamellar features that give rise to rigid areas with a high degree of molecular organization. Conversely, with ZIF-8H, its tetrahedral structures intertwine with collagen fibers, resulting in regions characterized by lower molecular order. The smaller size and significant chirality of the Zn(L-His)_2_ metal-organic structure are factors associated with the creation of entirely amorphous composites when combined with collagen, polyurethane, and polyelectrolyte. The influence of surface crystallinity will play a crucial role in evaluating the possible medical applications of these composites, as both their mechanical properties and biological interactions are contingent upon this surface attribute.

The effects of the physical reticulation induced by MOFs are noticeable in the mechanical stability of composite hydrogels and they compensate for the low chemical reticulation. Figure 5d shows the variation of the storage modulus as a function of the frequency. At a frequency of 1 Hz, the value of *G*’ was 17±0.1, 37±0.1, 40.6±0.1, and 32.5±0.1 Pa for H1, H2, H3, and H4, respectively. This enhancement in mechanical performance aligns with the inherent structural characteristics of the ZnMOFs. Specifically, the ZIF-8H structure significantly bolsters the storage modulus due to its tetrahedral nature, which results in the creation of amorphous surfaces within the hydrogel capable of withstanding greater deformation forces. This finding also suggests a correlation between the catalytic process involved in collagen fibrillogenesis, the formation of larger fibers, and improved mechanical resistance. The increased mechanical strength of hydrogels, particularly in the context of biomedical applications like wound dressings, warrants consideration for long-term usage. This consideration helps prevent phenomena such as cell-induced contraction and is particularly relevant for drug encapsulation, where substances like antibiotics, anti-inflammatories, and analgesics can positively influence the healing process.

**Figure 6.**
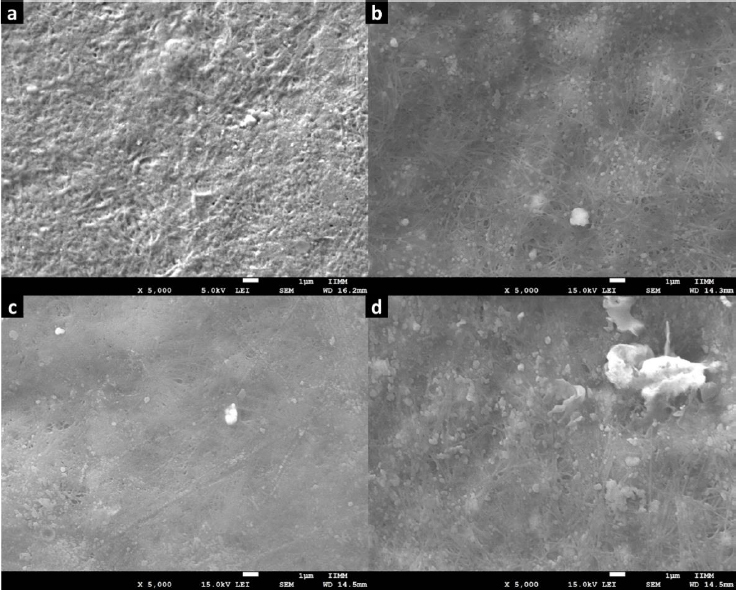
SEM image of (a) H1; (b) H2. (c) H3 and (d) H4.

Figure 6 shows the SEM images acquired for H1 and the composites H2-H4. For H1, a typical fibrillar morphology of collagen semi-IPN matrixes was observed; this microstructure exhibits intertwined fibers characterized by a standard interconnected porosity. Also, this microstructure was observed in the images of H2-H4. But, in these images, we observed some dispersed particles corresponding to Zn-MOFs. Furthermore, we observed some polymer agglomerates resulting from the physical reticulation produced by the MOF-polymer interaction. Regarding H3, it can observe the deposition of more pronounced and thicker fibers compared to the other microstructures. This observation aligns with the findings from the mechanical and turbidimetric analysis. In the case of H4, there is a greater presence of hemispherical aggregates, and this can be attributed to the high dispersion of the Zn(L-His)_2_ complex within the collagen-polyelectrolyte matrix. This high dispersion does not result in substantial crosslinking, allowing increased capacity to capture water molecules through hydrogen bonding.

**Figure 7.**
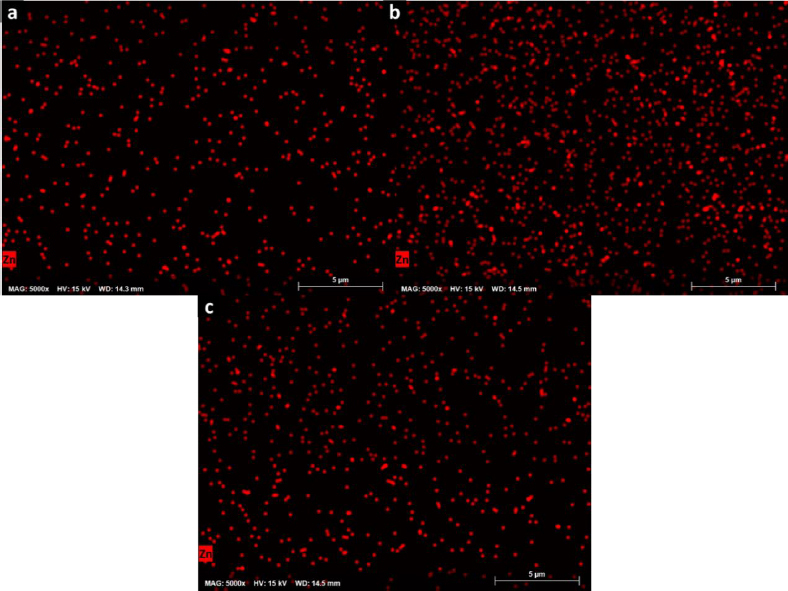
Zn composition maps acquired by EDX of (a) H2; (b) H3 and (c) H4.

The chemical homogeneity of composites was studied by EDX, and the composition maps are shown in Figure 7. They revealed a homogeneous distribution of Zn in the polymer matrix and a good dispersion of Zn-bioMOFs in the semi-IPN matrixes, contributing to obtaining reproducible properties in the cell scaffolds. In this context, these surfaces characterized by a consistent chemical composition possess distinctive surface characteristics. The abundant presence of zinc ions within the distributed MOFs can be harnessed for the regulation of cellular metabolism, modulation of cell signaling, enhancement of antibacterial properties, and promotion of catalytic effects in the secretion of crucial cytokines relevant to the healing process. Additionally, the adsorption capacity of these ZnMOFs can be leveraged for binding molecules of interest in the healing process, enabling precise and controlled drug dosing as previously reported.

### Evaluation of the biocompatibility in vitro

Zn^2+^ occurs in several fundamental metalloenzymes, which participate in the constitution and degradation of proteins, lipids, and nucleic acids, in the stabilization of protein structures, in control and regulation processes, and in the transfer of genetic information [27]. Zn salts are widely used as food supplements, and Zn deficiency is associated with impaired skin conditions. The influence of this element on skin functionality has been proven in clinical investigations [28]. Deters reported that Zn(L-His)_2_ and ZnSO_4_ increase the proliferation *in vitro* of keratinocytes. However, Zn(L-His)_2_ showed a more potent action with better tolerability and lower induction of differentiation [28]. From the above, it is expected that Zn-bioMOFs will benefit the biomedical performance of collagen-polyelectrolyte hydrogels.

The biocompatibility of H1 and the composites H2-H4 was evaluated by measuring the cell viability by the MTT assay using porcine fibroblasts, human monocytes, and RAW cells; these cells have primary functions during the healing process. Monocytes are responsible for activating signaling cascades for building and construction, macrophages are responsible for stimulating cells to build new collagen and blood vessels as well as protecting against infections, and fibroblasts are cells specialized in tissue reconstruction and remodeling. In this assay, 60 % of cell viability is considered the lowest permissible value to consider the material non-cytotoxic [29]. MTT reacts with the mitochondrial dehydrogenase enzymes of living cells and it is reduced to formazan. Therefore, this experiment allowed the detection of cellular stress in the hydrogels without considering the dead cells [30].

**Figure 8.**
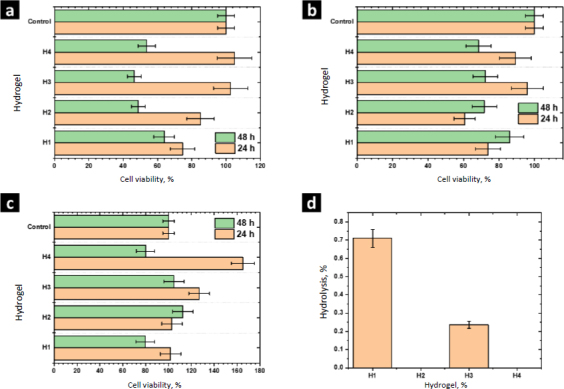
Viability of (a) porcine fibroblasts; (b) human monocytes and (c) RAW 264.7 cells; (d) Results for the hemolysis assay.

The viability of porcine fibroblasts cultured in contact with the hydrogels H1-H4 is shown in Figure 8a. The viability of fibroblasts cultured in contact with the hydrogel H1 was 75±7 %. Higher values of viability were observed culturing with the composites H2-H4. However, after 48 h of culturing, the viability of these cells decreased. The viability obtained with H1 and H4 was 63.8±6%. ZnMOFs tend to enhance the metabolic activity of fibroblasts in their respiratory function after a 24-hour culture period. To establish significance, a comparison is made between the values obtained for H1 and H4. This effect can be attributed to the Zn(L-His)2 complex, which, due to its smaller molecular size relative to L-1 and ZIF-8H, exhibits a more pronounced interaction with the biocomplex. This interaction positively impacts cellular metabolism. After 48 hours, the viability of fibroblasts within the composites containing ZnMOFs decreases, but it is important to note that this decrease is not indicative of cytotoxicity. Rather, it is associated with the adaptation of fibroblasts to the specific chemical composition dictated by each ZnMOF, resulting in a cessation of their accelerated metabolic activity.

The experiments performed with human monocytes and RAW cells showed similar results after 24 h of culture. Hydrogels H1-H4 were not cytotoxic and the composites H2-H4 showed higher cell viability than the matrix H1, showing that Zn-bioMOFs stimulate the cell metabolism of the cell lines evaluated in this study. In general, better results were observed with the composite H4, showing that the complex Zn(L-His)_2_ has better biological activity than MOFs L-1 or ZIF-8H. Currently, it is accepted that the biological activity of MOFs is due to the hydrolytic degradation of the coordination network, releasing metal ions and ligands, which are assimilated by cells. Due to structural complexity, MOFs are hydrolyzed slower than simple complexes such as Zn(L-His)_2_. Thus, the last one showed better performance at 24 h of culture.

After 48 h, the viability of human monocytes and RAW cells decreased. However, the values were higher than 60 %, showing the biocompatibility of these composites. At this time of culture, composites containing MOFs showed better performance than the composite H4 containing Zn(L-His)_2_. These results show that MOFs act as a controlled drug release system, dosing the hydrolysis products. Considering that the wound healing process requires a long time, MOFs could be better materials to prepare hydrogel wound dresses. These findings underscore the significance of surface and structural attributes in ZnMOFs for activating the metabolism of cells crucial to the healing process. In general, the Zn(L-His)_2_ complex stands out due to its amorphous surface and tendency to form agglomerates without precise molecular packing. This characteristic promotes short-term cellular interactions, leading to the stimulation of cell metabolism.

Results from the hemolysis assay are shown in Figure 8d. According to the criteria established by the ASTM-F756, all the evaluated materials are not cytotoxic (<2 % no hemolytic, 2-4 % slightly hemolytic, >4 % hemolytic). Further, we observed that L-1 and Zn(L-His)_2_ decrease the hemolytic character close to 0 %, showing the beneficial effects of these materials. The modest hemolytic potential observed in H3 is linked to the presence of thicker fibers and a higher storage modulus, factors that could potentially damage erythrocyte membranes. However, it is important to note that this level of hemolysis is not critical when considering applications involving contact with human blood.

**Figure 9.**
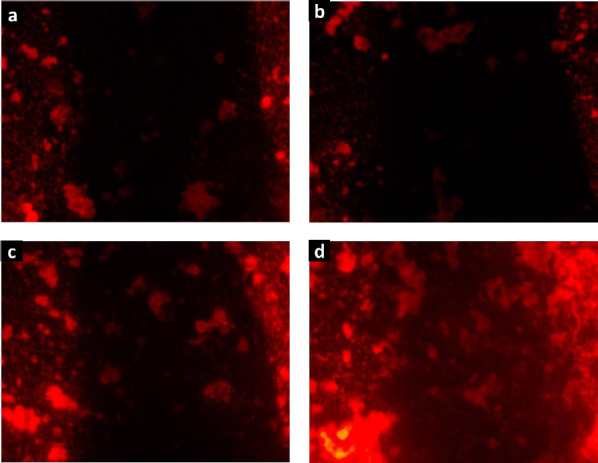
Proliferation assay carried out with RAW cells cultured in contact with composite hydrogels: (a) H1; (b) H2; (c) H3 and (d) H4.

The biocompatibility of hydrogels H1-H4 was confirmed by the cell proliferation assay. Figure 9 shows the fluorescence microscopy images of RAW cells stained with rhodamine B, which were cultured in contact with the degradation products of hydrogels. The images revealed large cell populations, being denser for H4. These findings align with the observations made using the MTT assay, suggesting that cells exposed to the degradation products of these collagen-polyurethane-polyelectrolyte-ZnMOFs composites do not exhibit cytotoxic effects that hinder cell growth and reproduction. The substantial bioavailability of the Zn(II) ion within the semi-IPN matrices is linked to this impressive biological performance, ensuring these composites function effectively in wound healing applications.

Cytokines are a diverse group of small proteins or glycoproteins that play a crucial role in cell signaling and communication within the immune system and other tissues in the body. They act as molecular messengers, transmitting information between cells to regulate various biological processes.

**Figure 10.**
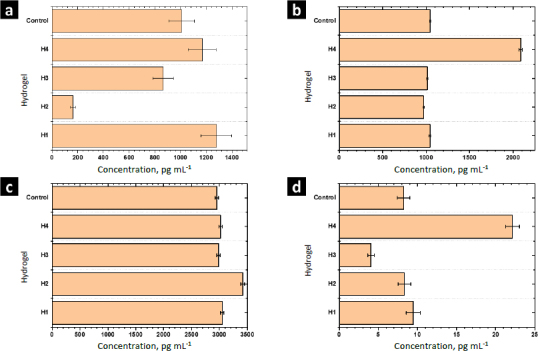
Cytokines evaluated by ELISA: (a) MCP-1; (b) TGF-β; (c) IL-10 and (d) TNF-α.

The function of cytokines includes regulating the immune response, inflammation, cell growth and differentiation, tissue repair, wound healing, and homeostasis. Figure 10 shows the results of the release from human monocytes of cytokines involved in the wound healing process: MCP-1, TGF-β, IL-10, and TNF-α.

MCP-1 is a potent monocyte attractant [31]. It has a vital role in the process of inflammation, where it attracts or enhances the expression of other inflammatory factors/cells [32]. Thus, it is very important to modulate the secretion of this cytokine during the wound-healing process. The concentration of MCP-1 for the control sample was 1006±100 pg mL^-1^. For H1 and H4 the concentration was higher, while it decreased for H2 and H3. Thus, MOFs L-1 and ZIF-8H do not have a positive effect on the modulation of this cytokine. Conversely, it is preferable to select the complex Zn(L-His)_2_ or not add any MOF to the polymer matrix. The heightened crystallinity of the semi-IPN matrix due to the interactions induced by ZIF-8H and L-1 in the polymer chains is correlated with a reduction in cell signaling responses in monocytes that lead to the secretion of MCP-1. This, in turn, would lead to a state of lower inflammation.

TGF-β increases the healing rate and the repaired tissue's breaking strength. It also enhances angiogenesis and consequent blood flow to dermal wounds. TGF-β reverses the negative effects of glucocorticoids on wound healing and thus may be useful in the treatment of chronic ulcers or wounds in patients whose normal responses have been impaired by therapy with steroids, radiation, or other drugs [33]. For the control sample (PBS-1X), the concentration of this cytokine was 1046±10 pg mL^-1^. For H1, H2, and H3 significant differences were not observed. Only H4 stimulated the secretion of TGF-β by human monocytes. The amorphous surface seen in H4, induced by the hydrophilic/hydrophobic interactions facilitated by Zn(L-His)2 within the collagen-polyelectrolyte chains, enables interaction with immune system cells, this, combined with its low level of crosslinking and high degree of swelling, plays a role in stimulating the secretion of TGF-β.

The concentration of IL-10 measured by ELISA was 2950±30 pg mL^-1^ in the control sample. A slight increase of IL-10 was observed with the hydrogel H1 and their composites H2-H4. The overexpression of IL-10 decreases the inflammatory response to injury, creating an environment conducive to regenerative adult wound healing, avoiding abnormal collagen deposition, and promoting the restoration of normal dermal architecture [34]. In this context, the composite featuring lamellar surfaces distributed within the fibrillar matrix has been observed to trigger the secretion of IL-10 in human monocytes. This signaling pattern is closely tied to the interactions that monocytes undergo within hydrophobic regions abundant in ZnMOF-containing aromatic rings.

Finally, the concentration of TNF-α found in the control was 9.4±0.9 pg mL^-1^. For H4 and H3, an increase and a decrease were observed concerning the control, respectively. For H1 and H2, significant differences were not observed. The inflammatory phase in wound healing is considered the preparatory process for the formation of new tissue. In this sense, TNF-α may have a beneficial role in wound healing, but it may have negative effects when overexpressed. Because an excess of TNF-α can inhibit the production of 3-hydroxyproline and, thus, the production of collagen [35]. The H3 material leads to a reduction in the secretion of this inflammatory cytokine. This effect is attributed to the strong fibrillar entanglement facilitated by the tetrahedral structures of ZIF-8H within the semi-IPN collagen-polyelectrolyte matrix, which promotes the inhibition of signaling pathways associated with TNF-α in monocytes. These findings illustrate that the unique surface properties of each composite, determined by the ZnMOF, enable the regulation of critical signaling pathways in the healing process. In this regard, H4 appears to stimulate the rapid formation of new tissue, while H3 helps control inflammation.

## Conclusions

The physicochemical characterization and the evaluation *in vitro* of the biocompatibility of the semi-IPN hydrogel (H1) and their composites (H2-H4) revealed an enhancement of the biomedical performance of the hydrogel matrix by adding Zn-MOFs. However, each MOF improves only some individual aspects of the performance. Zinc metal-organic structures (ZnMOFs) exhibit the ability to generate semi-crystalline composites when utilizing binders based on aromatic groups and amorphous composites when employing amino acids like L-histidine. These composites possess a fibrillar microstructure characterized by interconnected porosity, along with clusters containing ZnMOFs that influence their mechanical properties, swelling behavior, and crosslinking. The smaller the Zn compound coordinated with organic binders (Zn(L-His)_2_), the more effective it seems to be in activating the metabolism of critical cells involved in the healing process. Furthermore, the increased mechanical strength does not seem to compromise the hemocompatibility of the collagen-polyelectrolyte matrix. When considering degradation products, composites containing ZnMOFs do not seem to disrupt cell growth and proliferation in macrophages. In this context, H4 appears to promote rapid tissue regeneration, while H3 demonstrates the ability to regulate inflammation. Consequently, these innovative composite hydrogels hold promising potential for application in wound healing patches.

## References

[1] Meek S. T. Greathouse J. A. Allendorf M. D.. Metal-organic frameworks: A rapidly growing class of versatile nanoporous materials. Advanced Materials 23 (2011) 249-267. https://doi.org/10.1002/adma.201002854 10.1002/adma.201002854 20972981

[2] Singh N. Qutub S. Khashab N. M.. Biocompatibility and biodegradability of metal organic frameworks for biomedical applications. Journal of Materials Chemistry B 9 (2021) 5925-5934. https://doi.org/10.1039/d1tb01044a 10.1039/d1tb01044a 34259304

[3] Moharramnejad M. Ehsani A. Salmani S. Shani M. Makelshah R.E. Robatjazi Z.S. Parsimehr H.. Zinc-based metal-organic frameworks: synthesis and recent progress in biomedical application. Journal of Inorganic and Organometalic Polymers and Materials 32 (2022) 3339-3354. https://doi.org/10.1007/s10904-022-02385-y 10.1007/s10904-022-02385-y

[4] Bahrani S. Hashemi S. A. Mousavi S. M. Azhdari R.. Zinc-based metal-organic frameworks as nontoxic and biodegradable platforms for biomedical applications: review study. Drug Metabolism Reviews 51 (2019) 356-377. https://doi.org/10.1080/03602532.2019.1632887 10.1080/03602532.2019.1632887 31203696

[5] Bahr D. F. Reld J. A. Mook W. M. Bauer C. A. Stumpf R. Skulan A. J. Moody N. R. Simmons B. A. Shindel M. M. Allendorf M. D.. Mechanical properties of cubic zinc carboxyl- ate IRMOF-1 metal-organic framework crystals. Physical Review B 76 (2007) 184106. https://doi.org/10.1103/PhysRevB.76.184106 10.1103/PhysRevB.76.184106

[6] Abdelhamid H. N.. Zeolitic Imidazolate Frameworks (ZIF-8) for Biomedical Applications: A Review. Current Medicinal Chemistry 28 (2022) 7023-7075. https://doi.org/10.2174/1875533xmte2bmdqd0 10.2174/1875533xmte2bmdqd0 34102965

[7] Hoseinpour V. Shariatinia Z.. Applications of zeolitic imidazolate framework-8 (ZIF-8) in bone tissue engineering: A review. Tissue and Cell 72 (2021) 101588. https://doi.org/10.1016/j.tice.2021.101588 10.1016/j.tice.2021.101588 34237482

[8] Anderson S. L. Stylianou K. C.. Biologically derived metal organic frameworks. Coordination Chemistry Reviews 349 (2017) 102-128. https://doi.org/10.1016/j.ccr.2017.07.012 10.1016/j.ccr.2017.07.012

[9] Barbosa J. S. Figueira F. Braga S. S. Almeida Paz F. A.. Metal-organic frameworks for biomedical applications: The case of functional ligands in Metal-Organic Frameworks for Biomedical Applications. Mozafari Masoud Ed., Cambridge, USA, Woodhead Publishing, 2020, p. 69-92. https://doi.org/10.1016/B978-0-12-816984-1.00005-6 10.1016/B978-0-12-816984-1.00005-6

[10] Sun B. Bilal M. Jia S. Jiang Y. Cui J.. Design and bio-applications of biological metal-organic frameworks. Korean Journal of Chemical Engineering 36 (2019) 1949-1964. https://doi.org/10.1007/s11814-019-0394-8 10.1007/s11814-019-0394-8

[11] Wang H. S. Wang Y. H. Ding Y.. Development of biological metal-organic frameworks designed for biomedical applications: From bio-sensing/bio-imaging to disease treatment. Nanoscale Advances 2 (2020) 3788-3797. https://doi.org/10.1039/d0na00557f 10.1039/d0na00557f 36132764 PMC9418943

[12] Subramaniyam V. Ravi P. V. Pichumani M.. Structure coordination of solitary amino acids as ligands in metal-organic frameworks (MOFs): A comprehensive review. Journal of Molecular Structure 1251 (2022) 131931. https://doi.org/10.1016/j.molstruc.2021.131931 10.1016/j.molstruc.2021.131931

[13] Cai H. Huang Y. L. Li D.. Biological metal-organic frameworks: Structures, host-guest chemistry and bio-applications. Coordination Chemistry Reviews 378 (2019) 207-221. https://doi.org/10.1016/j.ccr.2017.12.003 10.1016/j.ccr.2017.12.003

[14] Kathalikkattil A. C. Babu R. Roshan R. K. Lee H. Kim H. Tharun J. Suresh E. Park D. W.. An lcy-topology amino acid MOF as eco-friendly catalyst for cyclic carbonate synthesis from CO_2_: Structure-DFT corroborated study. Journal of Materials Chemistry A 45 (2015) 22636-22647. https://doi.org/10.1039/c5ta05688h 10.1039/c5ta05688h

[15] Salama E. Ghanim M. Hassan H. S. Amer W. A. Ebeid E. M. El-Shazly A. H. Ossman M. Elkady M. F.. Novel aspartic-based bio-MOF adsorbent for effective anionic dye decontamination from polluted water. RSC Advances 12 (2022) 18363-18372. https://doi.org/10.1039/d2ra02333d 10.1039/d2ra02333d 35799940 PMC9215166

[16] Escamilla P. Viciano‐chumillas M. Bruno R. Armentano D. Pardo E.J. Ferrando‐Soria, Photodegradation of brilliant green dye by a zinc biomof and crystallographic visualization of resulting CO_2_. Molecules 26 (2021) 4098. https://doi.org/10.3390/molecules26134098 10.3390/molecules26134098 34279437 PMC8272194

[17] McKinlay A. C. Morris R. E. Horcajada P. Férey G. Gref R. Couvreur P. Serre C.. BioMOFs: Metal-organic frameworks for biological and medical applications. Angewandte Chemie 49 (2010) 6260-6266. https://doi.org/10.1002/anie.201000048 10.1002/anie.201000048 20652915

[18] Caldera-Villalobos M. Cabrera-Munguía D. A. Becerra-Rodríguez J. J. Claudio-Rizo J. A.. Tailoring biocompatibility of composite scaffolds of collagen/guar gum with metal-organic frameworks. RSC Advances 12 (2022) 3672-3686. https://doi.org/10.1039/d1ra08824f 10.1039/d1ra08824f 35425396 PMC8979324

[19] Mendoza-Villafaña J. J. Franco-Martínez M. G. Claudio-Rizo J. A. Cabrera-Munguía D. A. Caldera-Villalobos M. León-Campos M. I. Flores-Guía T. E. Cano-Salazar L. F.. Zn-based Metal-Organic Frameworks (MOFs) Incorporated into Collagen-Polysaccharide-based Composite Hydrogels for Their Use in Wound Healing. Asian Journal of Basic Science and Research 5 (2023) 41-54. http://doi.org/10.38177/AJBSR.2023.5106 10.38177/AJBSR.2023.5106

[20] Claudio-Rizo J. A. Rangel-Argote M. Castellano L. E. Delgado J. Mata-Mata J. L. Mendoza-Novelo B.. Influence of residual composition on the structure and properties of extracellular matrix derived hydrogels. Materials Science and Engineering C 79 (2017) 793-801. https://doi.org/10.1016/j.msec.2017.05.118 10.1016/j.msec.2017.05.118 28629082

[21] Mendoza-Novelo B. Mata-Mata J. L. Vega-González A. Cauich-Rodríguez J. V. Marcos-Fernández Á.. Synthesis and characterization of protected oligourethanes as crosslinkers of collagen-based scaffolds. Journal of Materials Chemistry B 2 (2014) 2874-2882. https://doi.org/10.1039/c3tb21832e 10.1039/c3tb21832e 32261482

[22] Caldera Villalobos M. Soriano Corral F. Claudio Rizo J. A.. Semi-interpenetración de matrices de colágeno-poliuretano con un polielectrolito biobasado. Avances en Ingeniería Química 4 (2022) 16-19. https://amidiq.com/avances-en-ingenieria-quimica/

[23] He J. Zhang G. Xiao D. Chen H. Yan S. Wang X. Yang J. Wang E.. Helicity controlled by the chirality of amino acid: Two novel enantiopure chiral 3D architectures containing fivefold interwoven helices. CrystEngComm 14 (2012) 3609-3614. https://doi.org/10.1039/c2ce25038a 10.1039/c2ce25038a

[24] Gu X. Zhou K. Feng Y. Yao J.. Morphology control of zeolitic imidazolate framework by addition of amino acid L-histidine. Chemistry Letters 44 (2015) 1080-1082. https://doi.org/10.1246/cl.150395 10.1246/cl.150395

[25] Materazzi S. Curini R. D’Ascenzo G.. Thermoanalytical behaviour of histidine complexes with transition metal ions. Thermochimica Acta 275 (1996) 93-108. https://doi.org/10.1016/0040-6031(95)02718-1 10.1016/0040-6031(95)02718-1

[26] Claudio-Rizo J. A. González-Lara I. A. Flores-Guía T. E. Cano-Salazar L. F. Cabrera-Munguía D. A. Becerra-Rodríguez J. J.. Study of the polyacrylate interpenetration in a collagen-polyurethane matrix to prepare novel hydrogels for biomedical applications. International Journal of Biological Macromolecules 156 (2020) 27-39. https://doi.org/10.1016/j.ijbiomac.2020.04.005 10.1016/j.ijbiomac.2020.04.005 32251751

[27] Chasapis C. T. Ntoupa P. S. A. Spiliopoulou C. A. Stefanidou M. E.. Recent aspects of the effects of zinc on human health. Archives of Toxicology 94 (2020) 1443-1460. https://doi.org/10.1007/s00204-020-02702-9 10.1007/s00204-020-02702-9 32394086

[28] Deters A. Schnetz E. Schmidt M. Hensel A.. Effects of zinc histidine and zinc sulfate on natural human keratinocytes. Forschende Komplementarmedizin und Klass. Naturheilkd 10 (2023) 19-25. https://doi.org/10.1159/000069903 10.1159/000069903 12624476

[29] León-Campos M. I. Claudio-Rizo J. A. Rodríguez-Fuentes N. Cabrera-Munguía D. A. Becerra-Rodríguez J. J. Herrera-Guerrero A. Soriano-Corral F.. Biocompatible interpenetrating polymeric networks in hydrogel state comprised from jellyfish collagen and polyurethane. Journal of Polymer Research 28 (2021) 291. https://doi.org/10.1007/s10965-021-02654-3 10.1007/s10965-021-02654-3

[30] Ferrari M. Fornasiero M. C. Isetta A. M.. MTT colorimetric assay for testing macrophage cytotoxic activity in vitro. Journal of Immunological Methods 131 (1990) 165-172. https://doi.org/10.1016/0022-1759(90)90187-Z 10.1016/0022-1759(90)90187-Z 2391427

[31] Yadav A. Saini V. Arora S.. MCP-1: Chemoattractant with a role beyond immunity: A review. Clinical Chimica Acta 411 (2010) 1570-1579. https://doi.org/10.1016/j.cca.2010.07.006 10.1016/j.cca.2010.07.006 20633546

[32] Singh S. Anshita D. Ravichandiran V.. MCP-1: Function, regulation, and involvement in disease. International Immunopharmacology 101 (2021) 107598. https://doi.org/10.1016/j.intimp.2021.107598 10.1016/j.intimp.2021.107598 34233864 PMC8135227

[33] Amento E. P. Beck L. S.. TGF-β and Wound Healing. Ciba Foundation Symposium 157-Clinical Applications of TGF-β, Bock G. R. Marsh J., Eds. Chichester, UK, John Wiley & Sons, 2007, p. 115-136. https://doi.org/10.1002/9780470514061.ch8 10.1002/9780470514061.ch8

[34] Peranteau W. H. Zhang L. Muvarak N. Badillo A.T. Radu A. Zoltick P.W. Liechty K.W.. IL-10 overexpression decreases inflammatory mediators and promotes regenerative healing in an adult model of scar formation. Journal of Investigation in Dermatology 128 (2008) 1852-1860. https://doi.org/10.1038/sj.jid.5701232 10.1038/sj.jid.5701232 18200061

[35] Rapala K.. The effect of tumor necrosis factor-alpha on wound healing. An experimental study. Annales Chirugiae et Gynaecologiae Supplementum 211 (1996) 1-53. https://europepmc.org/article/med/8790842 8790842

